# Regulation of iNOS on Immune Cells and Its Role in Diseases

**DOI:** 10.3390/ijms19123805

**Published:** 2018-11-29

**Authors:** Qingjie Xue, Yingchun Yan, Ruihua Zhang, Huabao Xiong

**Affiliations:** 1 Basic Medicine School, Jining Medical University, Jining 272067, Shandong, China; qjxue9797@126.com; 2 Precision Immunology Institute, Icahn School of Medicine at Mount Sinai, New York, NY 10029, USA; ruihua.zhang@mssm.edu; 3 Mental Health School, Jining Medical University, Jining 272067, Shandong, China; yanyc9797@163.com

**Keywords:** iNOS, T cells, macrophages, dendritic cells

## Abstract

In recent years, there have been many studies on the function of nitric oxide synthase (NOS) in experimental animals and humans. This review analyzes and explores the relationship between inducible nitric oxide synthase (iNOS) and T cells, macrophages, and dendritic cell et al. differentiation using data based on laboratory research, highlighting recent NOS laboratory research. Our insights into research prospects and directions are also presented.

## 1. Introduction

Nitric oxide (NO), which is synthesized by nitric oxide synthase (NOS), is the smallest known bioactive molecule, and can be produced by a variety of cells. NO plays an important role in neurotransmission, vascular function, host defense, and immune regulation [1]. Three NOS isoforms have been identified: neuronal nitric oxide synthase (nNOS), inducible nitric oxide synthase (iNOS), and endothelial nitric oxide synthase (eNOS) [2]. nNOS and eNOS are mainly expressed in neurons and epithelial cells, respectively, and are calcium-dependent. In contrast, calcium-independent iNOS can be produced by a variety of cells after induction by cytokines or other stimuli. NO is an important pro-inflammatory mediator with an effect on the immune system.

Indeed, NO plays a dual role in the process of immunoinflammation. On the one hand, NO can kill microorganisms and has a protective effect on the body, helping to fight against various viruses, such as herpes simplex virus (HSV). On the other hand, NO can damage normal tissue cells to generate pathogenic effects, and is widely involved in the development of various diseases, such as Borna disease [3]. According to existing research, macrophages and other effector cells, including neutrophils, monocytes, and endothelial cells, are the main effector cells involved in the antimicrobial effects of NO [4,5].

Studies have shown that iNOS is expressed by T cells, macrophages, and mature dendritic cells (mDCs), and regulates the differentiation and function of immune cells via nitration of key molecules involved in transcriptional or signaling pathways [6,7,8]. This article reviews and discusses recent experimental research.

## 2. iNOS and T Cell Differentiation

By inactivating and destroying infectious agents, NO is an important pro-inflammatory cytotoxic agent that protects the host against various pathogens [6,7,8,9]. Interestingly, NO also plays a key inhibitory role in immunity [10,11,12].

**Figure 1:**
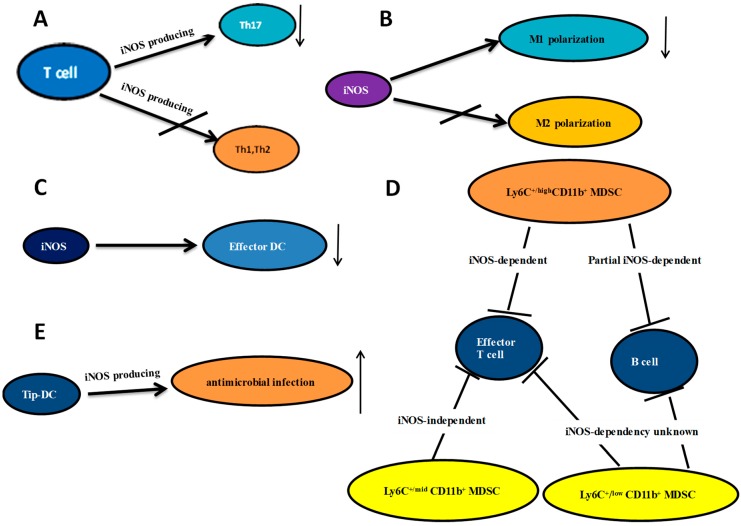
The relationship between iNOS and T cells, macrophages, dendritic cell, MDSC and Tip DC. (**A**) iNOS expressed by T cells negatively regulates Th17 differentiation without significant effects on Th1 or Th2 cells; (**B**) iNOS inhibits M1 polarization with no effect on M2 polarization; (**C**) The iNOS promotes effector DC differentiation; (**D**) MDSCs were sorted based on their cell surface expression of Ly6C and CD11b. The iNOS dependency of these relationships is listed; (**E**) Tip-DCs are important for antimicrobial infection; arrows ↓: decreasing; ↑: increasing.

Previously, we and other research groups reported that NO inhibits the production of IL-12 in dendritic cells and macrophages [13], which suggests that NO can control the production of Th1 immune responses by modulating IL-12 expression. It has also been reported that enhanced iNOS expression in T cells regulates the differentiation of Th17 cells. For example, in inflammatory bowel disease (IBD), iNOS expressed by T cells negatively regulates Th17 differentiation (Figure 1A) through nitration of orphan receptors γt associated with retinoic acid (RORγt) [14].

Our group has shown that iNOS^−/−^ mice exhibit enhanced Th17 cell differentiation without significant effects on Th1 or Th2 cells (Figure 1A). Expression of iNOS was found to be induced in activated CD4^+^ T cells, and the percentage of IL-17 produced by CD4^+^ T cells in WT (wide type) mice was significantly increased by treatment with the iNOS-selective inhibitor N6-(1-iminoethyl)-1-lysine dihydrochloride (L-NIL). In addition, NO donors, such as S-nitroso-N-acetylpenicillamine (SNAP), dose-dependently inhibit IL-17 production in cultured T cells from WT and iNOS^−/−^ mice [15].

Furthermore, nitration of the tyrosine residue of *RORγt* results in inhibition of RORγt-mediated IL-17 promoter activation, and naïve T cells obtained from iNOS-deficient mice induce more severe colitis in Rag^−/−^ mice than do normal control T cells. These results indicate that NO plays an important inhibitory role in controlling Th17 differentiation and highlight the importance of T cell-derived iNOS in mediating Th17-dependent immune responses. Th17 cells comprise a newer subset of the T helper cell family, and play a key role in the pathogenesis of autoimmune and inflammatory diseases [15,16]. Therefore, understanding the intrinsic inhibition program of Th17 cells will help in elucidating the mechanisms underlying the Th17 immune response and the development of inflammatory diseases, including IBD, multiple sclerosis (MS), and rheumatoid arthritis (RA). Mice carrying T cells from iNOS^−/−^ mice show a higher percentage of IL-17 produced by CD4^+^ T cells than do mice harboring T cells from WT mice [16].

These results indicate that iNOS derived from activated T cells selectively regulates T cell differentiation. Studies have also shown that NO can play a dual role in regulating immune responses [17]. In fact, NO produced by iNOS in macrophages and other innate immune cells is pro-inflammatory, and an essential component of the host immune response against various pathogens, including bacteria, parasites, and viruses [18].

Nonetheless, there is increasing evidence that NO can promote immunosuppression. We and other research groups previously reported a significant increase in IL-12 mRNA and protein expression in iNOS KO mice (control), suggesting that NO may inhibit IL-12-mediated Th1 immune responses [13,19]. Huang et al. [20] suggested that the enhanced Th1 immune response in iNOS knockout mice (iNOS^−/−^) after infection with *Leishmania major* is caused by an increase in IL-12 production by macrophages.

In a previous study, we clearly demonstrated that iNOS expressed by activated CD4^+^ T cells negatively regulates the differentiation of Th17 cells, consistent with findings that the NO donors NOC-18 and *S*-nitrosoglutathione (SNO) inhibit Th17 cell differentiation [11,21]. Therefore, the overall results support the view that by selectively inhibiting Th17 cell development, NO from iNOS controls T cell differentiation in activated T cells. The importance of *RORγt* in the development of Th17 cells has been well documented in mice. For instance, *RORγt^−/−^* mice fail to form lymph nodes or Peyer’s plaques, and their Th17 cells were severely impaired, suggesting that *RORγt* is the major transcription factor in Th17 cell differentiation [22].

Interestingly, *RORγt* expression in iNOS^−/−^ mouse CD4^+^ T cells cultured under Th17 conditions was comparable to that of CD4^+^T cells from WT mice, suggesting that enhanced Th17 cell differentiation is not a result of increased *RORγt* protein levels. In contrast, we found that the NOS donor SNAP inhibited *RORγt*-mediated *IL-17* promoter activation in a dose-dependent manner, which indicates that NO can control *RORγt* activity during *IL-17* gene transcription [13,15]. NO directly affects the activity of many proteins via tyrosine nitration [13].

Nitration of tyrosine residues in *RORγt* significantly impairs the binding of *RORγt* to the promoter region of the *IL-17* gene, inhibiting IL-17 transcription. Studies of *RORγt* mutants have shown that the tyrosine residue between amino acids 169 and 491 is a possible target for NO nitration [15]. Combining information based on the crystal structure of human *RORγt* and its ligand-binding domain using the antagonist digoxin, we found that several tyrosine residues are present in this region, with Tyr369 and Tyr382 located near the binding site. Thus, tyrosine nitration can greatly affect *RORγt* ligand formation and binding activity.

Moreover, mutation experiments have demonstrated that Tyr346 and Tyr359 of mouse *RORγt*, corresponding to Tyr369 and Tyr382 of human *RORγt*, play a key role in *RORγt* transcriptional activation. Therefore, Tyr346 and Tyr359 may be targets of NO in *RORγt* transcriptional modulation. Recently, Niedbala et al. [11] reported that the NO donor NOC-18 inhibits *AHR* protein expression in Th17 cells, and concluded that NO suppresses Th17 cell development by reducing *AHR* protein expression. However, under these conditions, we did not observe significant differences in AHR protein expression between CD4^+^ T cells from WT and iNOS^−/−^ mice, suggesting that *AHR* protein expression cannot explain the effect of iNOS produced by T cells on Th17 cell differentiation. A previous study showed that a tyrosine in IКBα is nitrated following activation of NOS, resulting in dissociation of IКBα from NF-κβ [6]. Other studies have stated that nitration of specific tyrosines in proteins may be structurally and functionally important [23], and we have reported a novel mechanism for modulating Th17 cell development through nitration of *RORγt* tyrosine residues. In addition, nitrosylation has been reported to regulate transcription factor activation [24,25]. For example, Khan et al. [26] reported that the NO donor SNO regulates NF-κB activation via S-nitrosylation of p65, which limits its binding activity. Th17 cells play a key role in the pathogenesis of several inflammatory diseases, including IBD and MS [27], and our future research will explore whether S-nitrosylation of *RORγt* is also involved in the regulation of IL-17 transcription.

We previously demonstrated that iNOS derived from T cells targets *RORγt*, resulting in impaired Th17 cell development. Transfer of naïve CD4^+^ T cells from iNOS^−/−^ mice into Rag^−/−^ mice induced more severe colitis, with an enhanced Th17 immune response. Furthermore, consistent with previous studies, iNOS^−/−^ mice develop more severe experimental autoimmune encephalomyelitis (EAE) than do wild type (WT) mice [11,28], though iNOS^−/−^ mice express significantly high levels of IL-17. This is consistent with the results of in vitro experiments in which iNOS transcripts are highly induced in T cells infiltrating the central nervous system (CNS) of WT mice, but not in iNOS^−/−^ mice with EAE. Interestingly, we found that compared with WT mice with EAE, iNOS^−/−^ mice with EAE exhibit a significant increase in CD4^+^ T cells producing interferon-γ(IFN-γ) and CD4^+^ T cells producing IL-17 [13].

In the EAE model, iNOS can be expressed by different cell types, including macrophages, dendritic cells, microglia, and T cells, yet it remains unclear which iNOS-expressing cells contribute to the regulation of Th1 cells [13,15]. In conclusion, iNOS expressed by activated T cells selectively regulates Th17 cell development, thereby controlling disease progression in colitis and EAE models. Collectively, iNOS is expressed by activated CD4^+^ T cells, and NO produced by iNOS can inhibit Th17 cell development in activated CD4^+^ T cells. Based on these observations, we proposed a new molecular mechanism for inhibiting the effect of NO on Th17 differentiation; iNOS expressed by T cells may play an important role in the development of inflammatory diseases by controlling the Th17 immune response [13].

## 3. The Effect of iNOS on Macrophages

NO can also inhibit the polarization of M1 macrophages, which can affect the differentiation and function of immune cells and contribute to the establishment of infection and the development of inflammatory diseases.

iNOS expressed by macrophages regulates the balance between M1 and M2 macrophages by modifying the transcription factor *IRF5* which, in turn, affects the development of IBD [29]. Toll-like receptor (TLR) ligands and inflammatory cytokines, including IFN-γ, can induce iNOS expression in many cell types, and it is clear that NO is an important pro-inflammatory cytotoxic agent that protects the host from various pathogens by inactivating and destroying infectious agents [18]. iNOS is a hallmark molecule of M1 macrophages, and we and other research groups reported that NO inhibits IL-12 production in dendritic cells and macrophages [13], suggesting that NO may control expression of molecules involved in innate immunity. Furthermore, iNOS-deficient mice are more susceptible to inflammatory diseases, such as EAE, than are WT mice [7,11]. Our previous study showed that iNOS-deficient mice exhibit enhanced M1 macrophage polarization (Figure 1B), with no effect on M2 macrophages [13]. Moreover, the iNOS-selective inhibitor L-NIL was shown to significantly enhance M1 macrophage polarization in cultured cells from WT mice.

Additionally, the NO donor SNAP inhibits M1 macrophage differentiation in cultured cells from WT mice and iNOS^−/−^ mice, and NO nitration of a tyrosine residue of *IRF5* inhibits M1 macrophage polarization. Systematic analysis has revealed a mutually inhibited loop that dynamically fine-tunes expression of iNOS and IL-12 in macrophages, and transferring iNOS-deficient macrophages into C57BL/6 mice resulted in a higher susceptibility to endotoxin shock. The results of these studies indicate that NO plays an important role in controlling M1 macrophage activation, and highlight the importance of myeloid-derived iNOS in regulating innate immune responses mediated by M1 macrophages [30].

Classical pathway-activated macrophages (M1) play an important role in host defense against pathogen infection, and are also involved in the pathogenesis of autoimmune and inflammatory diseases. Therefore, understanding the intrinsic regulatory processes of M1 macrophage differentiation will help in understanding the mechanisms that control innate immune responses and those involved in the development of human inflammatory diseases.

Furthermore, studies of endotoxin shock experimental models have shown that iNOS deficiency leads to more severe inflammation through M1 macrophage activation, indicating that iNOS derived from macrophages selectively regulates M1 macrophage differentiation. As stated above, iNOS is expressed by different cell types, including macrophages, dendritic cells, NK cells, and primary tumor cells [5], and NO in macrophages and other innate immune cells from iNOS^−/−^ mice is pro-inflammatory and an important component of the host immune response against a variety of pathogens [18]. Therefore, iNOS is an important marker of M1 macrophage activation. Despite increasing evidence that, in addition to its killing effect on pathogens, iNOS is involved in the regulation of immune responses, the importance of iNOS in controlling M1 macrophage differentiation is not fully understood. We previously demonstrated a significant increase in IL-12 mRNA and protein expression in iNOS^−/−^ mice, suggesting that iNOS contributes to macrophage-mediated regulation of pro-inflammatory cytokines [31].

In addition, Giordano et al. [31] have reported increased expression of inflammatory cytokines, including TNF-α, IL-6, IL-12, and IL-23, in iNOS^−/−^ mouse bone marrow-derived dendritic cells. Taken together, these results indicate that iNOS expressed by innate immune cells, including macrophages, can regulate the production of inflammatory cytokines, which are usually produced by M1 macrophages; thus, iNOS expressed by M1 macrophages plays a negative regulatory role in M1 macrophage differentiation.

This conclusion is supported by the following observations: (1) expression of characteristic M1 macrophage genes in iNOS^−/−^ mouse M1 macrophages is significantly increased, whereas defects in eNOS and nNOS have no effect on M1 macrophage differentiation; (2) iNOS^−/−^ mouse macrophages can significantly induce T cell activation; (3) the iNOS-selective inhibitor L-NIL can significantly increase expression of M1 macrophage characteristic genes, and the NO donor SNAP significantly inhibits M1 macrophage differentiation; (4) C57BL/6 mice transformed with iNOS^−/−^ mouse macrophages are more sensitive to endotoxin shock, accompanied by increased cytokine levels produced by M1 macrophages [30].

Therefore, our experiments support the notion that iNOS expressed by M1 macrophages regulates M1 macrophage differentiation by modulating the expression of characteristic genes in M1 macrophages. IL-10, an anti-inflammatory cytokine that reportedly inhibits macrophage function and controls inflammation, can be produced by a variety of cell types, including macrophages, T cells, and B cells. M2 macrophages also produce IL-10 which, in turn, leads to modulation of the immune response. To rule out the possibility that enhanced M1 macrophage differentiation is due to IL-10 in iNOS^−/−^ mice, we examined IL-10 expression in WT and iNOS^−/−^ mice macrophages under M1 conditions and found that IL-10 mRNA was similarly expressed.

In addition, the NO donor SNAP significantly inhibits expression of M1 macrophage marker genes. This indicates that enhancing the differentiation ability of M1 macrophages in iNOS^−/−^ mice does not depend on the action of IL-10. *IRF5* is a member of the interferon regulatory factor (*IRF*) family and has a variety of functions, including activation of genes encoding inflammatory cytokines, type I interferons, and tumor suppressors [14,32]. Since M1 macrophages express high levels of *IRF5* and overexpression of *IRF5* in M2 macrophages induces full expression of M1 macrophage marker genes, *IRF5* has been recognized as a key transcription factor for M1 macrophage differentiation [33]. Furthermore, *IRF5*-deficient mice are resistant to lethal endotoxic shock. Evidence suggests that activation of *IRF5* expression determines the development of the M1 macrophage lineage.

Interestingly, microarray analysis has shown that *IRF5* expression in iNOS^−/−^ mouse M1 macrophages is comparable to that in WT mouse M1 macrophages. Furthermore, SNAP or L-NIL has no significant effect on *IRF5* protein expression in WT and iNOS^−/−^ mouse M1 macrophages, suggesting that enhancement of M1 macrophage differentiation is not a result of increased *IRF5* protein levels. Regardless, knockdown of *IRF5* in iNOS^−/−^ mouse M1 macrophages reduced expression of M1 marker cytokines, suggesting that iNOS can control *IRF5* activation during M1 macrophage differentiation. Nitration of RORγt tyrosine residues significantly impairs the binding of *RORγt* to the promoter region of the *IL-17* gene, suppressing IL-17 transcription, and this may also explain the observed regulation of *IRF5* [15].

Indeed, NO produced by iNOS can nitrate a tyrosine residue of the *IRF*5 protein in M1 macrophages of WT mice, but not in iNOS^−/−^ mice. Furthermore, chromatin immunoprecipitation (ChIP) analysis has revealed that *IRF5* DNA-binding activity is significantly enhanced in iNOS^−/−^ mouse macrophages, and that SNAP inhibits this activity. Furthermore, we identified that residues Tyr74 and Tyr104 of *IRF5* are important for IL-12 promoter activation. Previous studies have shown that a tyrosine of *IkBa* is nitrated after NO synthase activation, resulting in dissociation of IkBa from NF-kB, and other studies have shown that nitration of specific tyrosines in proteins may have structural and functional significance [26,34].

In summary, our previous study revealed a novel mechanism for regulating M1 macrophage differentiation via nitration of *IRF5* tyrosine residues. M1 macrophages have been implicated as a key player in host defense against bacterial infections, endotoxic shock, and tumor growth [34,35,36,37], and it is well known that iNOS-derived NO helps to directly kill a variety of pathogens. Nonetheless, the mechanism by which iNOS regulates the differentiation of M1 macrophages, in vivo, remains unknown. We have found that iNOS-deficient mice are sensitive to *L. monocytogenes* infections, as NO is directly involved in killing bacteria, whereas expression of M1 macrophage marker molecules was found to be significantly increased in iNOS-deficient mice after infection, indicating that M1 macrophage activity was enhanced in iNOS-deficient mice during *L. monocytogenes* infection [30].

Consistent with these results, mice transplanted with iNOS^−/−^ macrophages were sensitive to lethal endotoxin shock, and expression of M1 macrophage marker molecules was enhanced. Thus, iNOS expressed in macrophages plays a negative role in M1 macrophage differentiation in vivo, even though it is a key marker of M1 macrophages. In another in vivo model of melanoma vaccination, L-NIL treatment significantly reduced tumor size in tumor-bearing mice, and tumor-infiltrating cell analysis showed that treatment with this selective iNOS inhibitor significantly increased the percentage of M1 macrophages in the tumor microenvironment [30]. Therefore, iNOS inhibition in the tumor microenvironment promotes M1 macrophage differentiation, resulting in tumor reduction; this further confirms that NO can negatively regulate M1 macrophage differentiation in vivo. Overall, analysis of a series of experimental results shows the dynamic balance of innate immune regulation.

Similarly, results of our previous study provide the initial evidence that macrophages have similar dynamic circuits capable of fine-tuning expression of iNOS and IL-12. Taken together, our studies clearly demonstrate that, in addition to acting as a signature marker for M1 macrophages, M1 macrophage-expressed iNOS dedifferentiates these cells. Based on these experiments, we propose a new molecular mechanism, whereby the effect of NO produced by iNOS on M1 macrophage dedifferentiation involves inhibition of *IRF5* activation [33,38,39,40,41]. The findings establish a new view that iNOS expression in macrophages not only selectively regulates *M1 macrophage* gene expression, but also regulates M1 macrophage dedifferentiation, leading to the control of innate immune responses.

## 4. Regulation of Dendritic Cells Differentiation by iNOS

Recent studies have found that NO produced by upregulated iNOS after mDC activation controls the balance of effector DCs and regulatory DCs, which is accomplished by inhibiting NF-κB signaling and inflammatory body activity [42,43,44]. We found that although the number of CD11c^+^ CD11b^+^ effector DCs producing IL-12, TNF-α, and IL-6 in iNOS^‒/‒^ mice to be increased, the percentage of CD11c^+^CD11b^+^ regulatory DCs expressing IL-10 and PD-1 was the same as that of wild type mice. In vitro studies further confirmed that effector DCs elevated cell differentiation in iNOS^‒/‒^ mice, and that the protein and mRNA levels of the corresponding molecules increased [45].

In addition, Lipopolysaccharides (LPS)/IFN-α stimulates WT bone marrow dendritic cells (BMDCs), and results in increased iNOS expression. Furthermore, the iNOS-specific inhibitor L-NIL (dihydrochloride) selectively promotes effector DC differentiation (Figure 1C), and mimics the results observed in iNOS^‒/‒^ mice [46]. Conversely, the NO donor SNAP (soluble NO attachment protein) significantly inhibits the developmental differentiation of effector DCs. We also found that the maturation and differentiation of more effector DCs from iNOS^‒/‒^ mice results in an increase in T cell activation and proliferation [46]. In vivo experiments showed that iNOS^‒/‒^ mice with digestive tract perfusion of *Citrobacter rodentium* exhibited more severe symptoms of colitis and marked inflammatory cell colonic infiltration compared with WT mice [47].

In addition, the number of effector DCs in the spleen and colon of iNOS^‒/‒^ mice was significantly increased, compared with their numbers in WT mice after perfusion of *C. rodentium,* and we found that by inhibiting the NF-κB pathway, the function of NO-regulated DCs produced by iNOS is enhanced after mDC activation. This result shows that mDC-derived iNOS can negatively regulate the development of effector DCs [48]. The above experimental results allow for possible examination of the differentiation and maturation of plasmacytoid dendritic cells (pDCs), and their role in disease processes.

Effector DCs secrete cytokines, such as IL-12, TNF, IL-6, and IFN-γ, to induce an adaptive immune response, including promoting T cell differentiation into Th1, Th2, or Th17 subpopulations, which can favor pathogen clearance; however, cytokines can cause tissue damage. For example, iNOS^−/−^ mice showed enhanced maturation and differentiation of effector DCs after LPS and IFN-γ stimulation. Similarly, levels of molecules associated with effector DCs, including IL-12, IL-23, IL-1β, and TNF, but not those associated with regulatory DCs, such as IL-10 and PD-1, were increased in BMDCs cells from iNOS^−/−^ mice [48]. Therefore, iNOS mediates NO production, which specifically inhibits the differentiation of effector DCs.

In many cell types, iNOS can be induced by cytokines and other stimuli, and iNOS activity has been demonstrated in a variety of cells and tissues [49,50]. In contrast, nNOS and eNOS are present in cells as preformed proteins with activity that is linked to an increase in intracellular Ca^2+^ and binding of calmodulin in response to a neurotransmitter or vasoactive substance.

There are also additional levels of regulation for all three NOS subtypes that may participate during the immune response. The NOS isoforms invoked by cytokines or other stimuli in phagocytic, dendritic, NK, T cells, and B cells, is mainly iNOS [4,46]. In immune cells, iNOS can be induced in a non-calcium-dependent manner by a variety of stimuli, including LPS, IFN-γ, and TNF [3,50]. Moreover, iNOS-deficient mice display an increased effector DC cell phenotype, including a population of cells expressing IL-12, TNF, and IL-6. Following LPS and IFN-γ stimulation, iNOS deficiency results in high expression of mouse MHC-II and costimulatory molecules CD80 and CD86 in BMDCs, conferring the ability to present antigens and activate CD4^+^ T cells on these DCs.

Overall, the inhibitory effect of NO on effector DC differentiation may be due to its inhibition of NF-κB signaling and inflammatory molecule activation. iNOS^−/−^ mice with oral *C. rodentium* bacterial infection develop more severe colitis than do WT mice, harboring a population of DCs with significant amplification effects in their spleen and colon [51,52]. These results indicate that NO produced by DCs, and iNOS in DC, are associated with increased pathogenicity in the inflammatory response. Similar results were observed in an endotoxin-induced sepsis model in which DC-derived iNOS negatively controlled effector DC development. Intrinsic DCs require inhibition of effector DC differentiation and exhibit limited inflammatory CD4^+^ T cell responses. DC-derived NO inhibits activation of NF-κB signaling and inflammation. These results suggest that targeting DC-intrinsic iNOS may be an effective treatment strategy for immune and inflammatory diseases. Therefore, our previous research provides the basis for targeting DC-intrinsic iNOS as a therapeutic strategy against autoimmune and inflammatory diseases.

## 5. Important Role of iNOS in MDSCs and Tip-DCs

Myeloid-derived suppressor cells (MDSCs) are immature heterogeneous populations of myeloid cells (IMCs), including precursors of dendritic cells, granulocytes, and macrophages [53]. MDSCs express high levels of iNOS-mediated nitrification of signal transducer and activator of transcription 1(STAT1) to block IFN-γ signaling [54,55]. Therefore, iNOS is an important mediator in the suppressive function of MDSCs (Figure 1D). iNOS produces NO in myeloid cells to inhibit the defense mechanisms of tumor cells or invading microorganisms. In mice with tumorigenesis, high expression of iNOS is a marker of MDSCs. NO is used by MDSCs to nitrate the T cell receptor, and STAT1 to suppress T cell activation and promote antitumor immune responses [54,56].

MDSCs can also directly alter the pathogenesis of microorganisms to suppress immune cells [54,56]. For example, NO has antimicrobial and antiparasitic activities [55], and NO production during virus infection can inhibit viral replication and invoke an immune response [56,57]. As inflammatory myeloid cells might not necessarily have a suppressive function, the suppressive capacity of MDSCs was analyzed in vitro. It has been reported that NO secretion by immature Ly6G^+/^CD11b^+^ myeloid cells can suppress the proliferation of cells isolated from the lymph node in models such as leishmaniasis [57] and trypanosomiasis [58] infection. In addition, by increasing the abundance of regulatory T cells, MDSCs participate in immune reaction suppression [59,60]. In clinical application, blocking the suppressive mechanism(s) of MDSCs may be an attractive approach for MDSC depletion. 

In 2004, a new DC subset was discovered that exhibits iNOS/NO and TNF production during *L. monocytogenes* infection [61]. Due to the high levels of co-stimulatory molecules produced by presenting DCs, prime alloreactive T cells do not adhere, but have a morphology similar to that of DCs; these TNF/iNOS-producing DCs were called Tip-DCs [62]. Tip-DCs are important for antimicrobial infection [63] (Figure 1E); Tip-DC accumulation was also found in the liver of mice infected with *Trypanosoma brucei* [64]. Other data have shown that human Tip-DCs are able to prime naïve T cells, promoting Th1 cell differentiation in vitro [65]. Thus, Tip-DCs may be involved in polarizing naïve T cells. Nevertheless, iNOS expressed by TipDCs is important for the defense of the host against the obligatory intracellular pathogen *Leishmania major* [66]. Additionally, it has been reported that Tip-DCs produce IFN-β, which is helpful for healing of leishmaniasis [67].

Tip-DCs are positive for Ly6G and/or Ly6C, which are known to be expressed by neutrophils, DCs, and immature monocytes [63,68]. In a murine model of *T. brucei* infection [68,69], CCR2^+^ monocytic cells derived from Ly6C^−^- and CD11b^−^-expressing cells were believed to represent the Tip-DC precursor. In the model, differentiation to Tip-DCs was independent of MyD88 and IFN-γ signaling, whereas IFN-γ stimulation was necessary for the production of iNOS/NO [69]. Thus, it is obvious that TiP-DCs can originate from CCR2^+^ Ly6C^high^ macrophage/DC progenitor (MDP) cells, and can produce NO after appropriate stimulation [68].

## 6. The Role of iNOS in Cancer and Cancer Immunotherapy

In cancer, the role of NO in immune function may be contradictory. NO represents the mechanism by which M1-polarized macrophages kill tumors, but it has also been found in many cancer models to drive tumor-mediated immunosuppression [70,71]. This seemingly contradictory phenomenon indicates that the disparate production of NO and expression of NO-producing enzymes, such as iNOS, may bias the balance toward immunity or immunosuppression, depending on the cell type. We found that the antagonistic balance between MDSC and Tregs limits the beneficial effect of iNOS inhibition on tumor immunity, but that it can be overcome by targeting Tregs simultaneously with an iNOS inhibitor and low-dose cyclophosphamide [72]. We believe that iNOS inhibition enhances accumulation of Tregs in tumor-bearing mice, and a combination therapy strategy for MDSC and Tregs can be applied, which may lead to significant recovery of the body’s immune function. Indeed, this has been explored in a study of a therapeutic method that has significant antitumor activity in a melanoma mouse model [72].

## 7. Conclusions and Future Prospects

iNOS function not only contributes to pathogen killing, but also has immune-regulatory effects, such as inhibiting T cell activity. Many types of immune cells express iNOS, and iNOS can also be expressed by nonimmune cells, such as endothelial cells, fibroblasts, and keratinocytes [1].

However, it is unclear whether all immune cells express iNOS; in particular, the functions and mechanisms of iNOS produced by immune cells remain unclear, especially regarding the regulation of immunity and tolerance regulation of autoimmune and inflammatory diseases. In addition, it is not clear whether ILC cells express iNOS, and what the function of NO would be in these cells.

Of particular concern is that pDCs plays a crucial role in the regulation of the immune response and immune-related diseases. Furthermore, it remains unclear whether iNOS regulates the differentiation and function of pDCs, and participates in the development of infection and inflammatory diseases. Future studies should investigate whether other immune cells, such as pDCs, also have a nitration-regulating function, whether iNOS regulates pDC expression and the developmental differentiation and function of these cells, and whether iNOS-mediated regulation of pDC expression can be extended to the treatment of inflammatory diseases. Additionally, how iNOS expressed by various immune cells regulates their differentiation and function remains unclear, and future studies should provide more in-depth data to support the molecular regulation of immune cell differentiation and function. Based on previous studies, the mechanism by which pDC-derived iNOS negatively regulates pDC differentiation and function should be further explored in an in-depth study to understand the specific mechanism of action involved in the development of inflammatory diseases.

Future studies will focus on molecular mechanisms, for example, how iNOS-derived NO regulates various transcription factors expressed by immune cells. Exploring the functions of iNOS produced by nonimmune cells is also important.
